# Effect of viscoelastic polymer on damping properties of precast concrete panel

**DOI:** 10.1016/j.heliyon.2021.e06967

**Published:** 2021-05-05

**Authors:** Khemapat Tontiwattanakul, Jirawin Sanguansin, Vatanavongs Ratanavaraha, Vanchai Sata, Suchart Limkatanyu, Piti Sukontasukkul

**Affiliations:** a Sound and Vibration Engineering Research Group, Department of Mechanical and Aerospace Engineering, Faculty of Engineering, King Mongkut's University of Technology North Bangkok, Thailand; b Construction and Building Materials Research Center, Department of Civil Engineering, Faculty of Engineering, King Mongkut's University of Technology North Bangkok, Thailand; c School of Transportation Engineering, Suranaree University of Technology, Nakhon Ratchasima, Thailand; d Sustainable Infrastructure Research and Development Center, Depertment of Civil Engineering, Faculty of Engineering, Khon Kaen University, Khon Kaen, Thailand; e Department of Civil Engineering, Faculty of Engineering, Prince of Songkla University, Hat Yai, Songkhla, Thailand

**Keywords:** Precast concrete, Viscoelastic polymer, Damping, Sound insulation

## Abstract

Precast concrete system has been widely used in modern day constructions due to its high efficiency in both production time and cost. However, because of the way it is constructed (with flat and dense surface), problems with sound reflection and transmission often exist. It is known that increasing of damping property of materials can reduce the transmission of impact sound and vibration which could lead to an improvement in sound insulation performance. In this study, a type of Viscoelastic Polymer Sheet (VPS) was introduced and attached to concrete precast panels with an aim to improve damping property of precast concrete panels. Seven precast concrete specimens with various patterns and attachment position of VPS were prepared. Effect of patterns and locations of attaching VPS on damping property are investigated and discussed.

## 1 Introduction

As population growth and urbanisation continue, the demand of housing near cities has been increasing significantly. More people find themselves living in apartments or condominiums instead of traditional houses. However, there are quite a number of problems when it comes to living in an apartment block, especially when it is built in cubical form with precast concrete material. Common problems that arise are related to temperature and sound. In the case of thermal problems, the utilization of materials with high thermal insulation properties such as lightweight concrete, cellular concrete panel, or concrete panels containing thermal capture agencies (i.e., Phase change materials) can be used to ease out and provide comfort [1, 2, 3, 4].

In the case of sound properties, an attention has been paid to the application of viscoelastic polymer materials (VPM) for vibration control. The applications of VPM to reduce vibration and sound induced vibration can be found in several industries. For example, the VPM sheet was used to reduce vibration happened to a steel wall structures of oil rig building [5]. A sheet of VPM (polyurethane and acrylic type) with thickness of approximately 2 mm was attached to the wall to reduce the vibration from sound waves. In the aircraft industry, sound protection in the cabin was be achieved by lining viscoelastic polymer sheets to the wall around the cabin to control the vibration and noise generated by various parts of an aeroplane [6]. VPM is excellent in dissipating vibration by behaving like viscous fluid [7]. The energy generated from the deformation of the material does not accumulate in the material itself but dissipate as heat loss and reduce the kinetic energy of the system.

However, in the field of civil engineering, investigations related to damping are focused mostly on structural components subjected to vibration, seismic, cyclic or fatigue loadings. Only a few number of studies has been investigated on the use of VPM to improve damping and sound properties. For example, Lee et al. [8] proposed a technique to improve in damping property of a composite that made of preplaced aggregates and polyurethane matrix. In a similar kind of work, Lee at al [9]. also introduced a new type of prepacked concrete which incorporated coarse aggregates coated with polyurethane that is capable to increase the damping property by about 10% under the flexural vibration.

Since there are very few studies related to the application of VPM sheet to improve damping and sound properties of concrete material, and most of which are focused on using coated aggregates or rubber particles as a part of concrete constituent ingredients, therefore there is obviously a lack of knowledge on the direct application of VPM attached to the surface of concrete panels. This paper aims to propose an alternative technique to increase the damping property of the precast concrete panel by using VPM sheet (VPS). Seven precast concrete panels were built as the specimens. VPS strips were attached to the specimens in different patterns and different surface constrain. Damping lost factor is measured and calculated in according to the guideline given by ASTM E756 and C215.

## 2 Objective of this study

**Figure 1:**
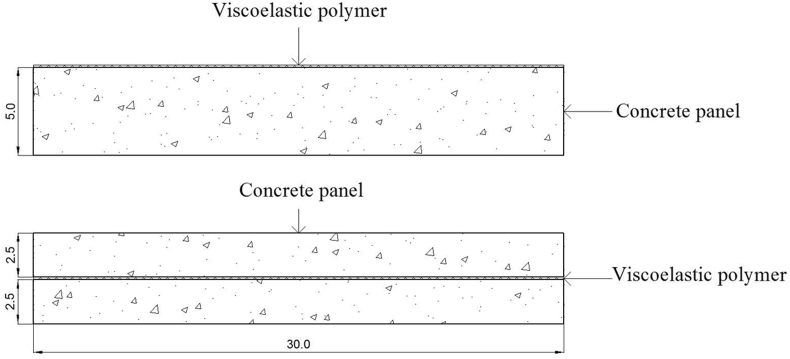
Location of VPS: Surface attachment (top) and Mid-plate insertion (bottom).

The aim of this study is to find a use of VPS to increase the damping loss factor by strategically attach VPS to precast concrete panels. VPS referred to in this study was a type of high adhesion butyl rubber adhesive tape coated with aluminum film. There two surface constraint conditions were introduced; 1) surface attachment - VPS was attached directly on the surface of the specimens to provide a free damping condition and 2) Mid-plate insertion - a VPS was inserted at the middle layer of the specimens to provide a constrained layer (see Figure 1).

**Figure 2:**
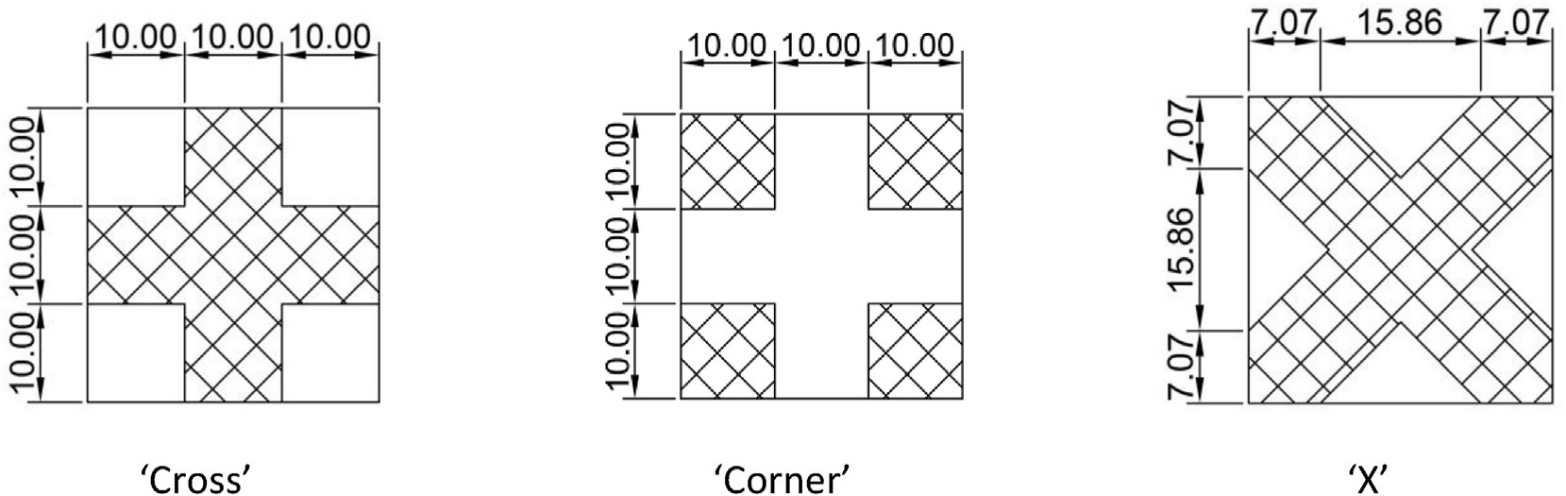
Pattern of VPS attachment.

Apart from the surface constraints, the pattern of attachment is also considered. Using the information obtained from the next section (Section 3: Mode shape analysis), the VPS was cut and attached to the specimens in three patterns - namely ‘Cross’, ‘Corner’, and ‘X’ - as shown in Figure 2. It can be seen that the three patterns cross the nodal lines of the mode shapes that yield to constrain the vibration.

## 3 Mode shape analysis

**Figure 3:**
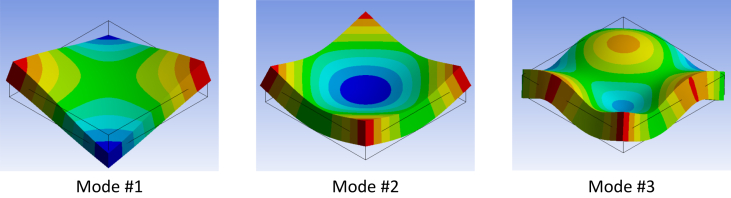
The first three mode shapes (bending) of a square plate.

Vibration of a precast panel in any conventional shape such as rectangular plates can be analytically studied via the theory of plates [10]. The equation of motion of rectangular plates is given by

(1) $$\frac{\partial^{4}W}{\partial x^{4}}+2\frac{\partial^{4}W}{\partial x^{2}\partial y^{2}}+\frac{\partial^{4}W}{\partial y^{4}}+\frac{\rho h}{D}\frac{\partial^{2}W}{\partial t^{2}}=\frac{p_{0}\left(x,y,t\right)}{D}$$

where *W* is the displacement of the vibration of the plate, ρ is the bulk density, h is the plate thickness, and $p_{0}$ is the excitation force. D is the bending rigidity of the plate is given by

(2) $$D=\frac{Eh^{3}}{12\left(1-\nu \right)}$$

where ν is poisson's ratio and E is Young's modulus. Once the dimension of a rectangular plate and the boundary condition is given, the solution of (1) can be given by mean of natural frequencies and mode shapes. It should be noted that Eq. (1) is undamped thus the damping ratio cannot be analytically obtained. By considering free-free-free-free boundary condition, the modal frequencies of a square plate can be calculated by

(3) $$f_{nm}=\frac{\lambda_{nm}^{2}}{2\pi a^{2}}\sqrt{\frac{Eh^{3}}{12m_{s}\left(1-\nu^{2}\right)}}$$

where $f_{nm}$ is the modal frequencies, $\lambda_{nm}$ is the wavelength of the mode shapes, n and m are the modal indices, a is the length of square sides, and is $m_{s}$ the surface density of the plate [10]. The mode shapes of the plate can also be analytically calculated. Alternatively, the mode shape can also be computed numerically. Figure 3 shows the mode shape of the first three bending modes calculated by ANSYS software. The model represents a concrete plate with dimension of 30 cm × 30 cm x 5 cm. The boundary condition is free-free-free-free and the mesh size in the calculation is set to be 20 times smaller than the dimension of the model to capture the mode shape. It should be noted that each mode will have corresponding nodal line(s), where is the position on the plate that completely does not move.

**Figure 4:**
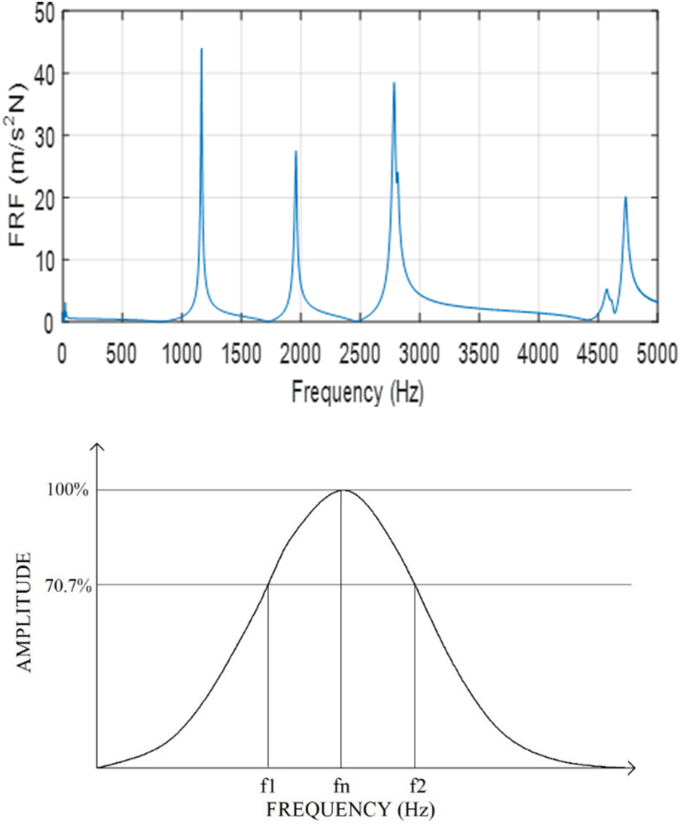
Measured FRF (top) and determination of the half power point (bottom).

In practice, the damping ratio of a square plate can be measured by applying the modal testing. In according to ASTM E756, the half power method is applied to determine the damping ratios once the frequency response function (FRF) of a plate is measured. The left side of Figure 4 shows an FRF of a square plate, denoted by A(ω), which is given as the ratio of Fourier spectrum of the acceleration signal $\ddot{X}\left(\omega \right)$ to excitation force F(ω)

(4) $$A\left(\omega \right)=\frac{\ddot{X}\left(\omega \right)}{F\left(\omega \right)}$$

The FRF A(ω) can be decomposed into a series of single degree of freedom systems thus the damping ratio can be obtained by windowed out each individual peaks (see the right hand side of Figure (2)) and find the half power frequencies $f_{1}$ and $f_{2}$, which is frequencies that have half of power w.r.t the peak that indicates the modal frequency or natural frequency $f_{n}$. The damping ratio ζ can be computed by

(5) $$\zeta =\frac{\left(f_{2}-f_{1}\right)}{2f_{n}}$$

However, an alternative index so-called damping loss factor η is widely used in industry. The relation between the two is η=2ζ. The damping loss factor will be to indicate the effect of the damping throughout this work. The damping loss factor indicates the ability of structure to dissipate energy. The higher the damping loss factor allows for the faster the decaying of vibration.

## 4 Experimental part

### 4.1 Specimens preparation

There were seven precast concrete with the dimension of 300 × 300 × 50 mm were prepared as the test specimens. The mix proportion of concrete was set at 1.00/0.62/2.47/3.00 by weight (cement/water/fine/coarse). The mixing process began by dry mixing cement and sand, later the water was added to form mortar slurry. Lastly, coarse aggregates were added and the mixing continued for further 1–2 min. The specimens passed the mechanical property test in according to the international standard such as compressive strength (BS 1881 part 4), unit weight of fresh concrete (ASTM C138), slump of fresh concrete (ASTM C143), water absorption and density of concrete (ASTM C642) and the results on concrete properties are given in Table 1.

**Table 1:** Properties of concrete.

Property	Results	Unit
Unit weight of fresh concrete	2,357	kg/m^3^
Slump	75	mm.
7-day Compressive strength	25	MPa
28-day Compressive strength	35	MPa
Dry density	2,213	kg/m^3^
Water absorption	5.02	%

The fresh mixed concrete was transferred into molds and compacted by a vibrating table the were covered by plastic sheets for more than 24 h. After that, the specimens were removed from the mold and wrapped with plastic sheets and placed in under the room temperature for more than 28 days. The specimens then were attached with the VPS resulting in 6 combinations from the three attachment patterns and two surface constraint conditions as described in Section 3. The specimens and their description are listed in Table 2.

**Table 2:** Description of the specimens.

No.	Specimen	Description
1	Control	No damping treatment
2	SA-Cross	PVS attached in ‘cross’ pattern on the top surface
3	SA-Corner	PVS attached in ‘corner’ pattern on the top surface
4	SA-X	PVS attached in ‘X’ pattern on the top surface
5	MPI-Cross	PVS attached in ‘cross’ pattern in the middle
6	MPI-Corner	PVS attached in ‘corner’ pattern in the middle
7	MPI-X	PVS attached in ‘X’ pattern in the middle

### 4.2 Measurement of damping loss factor

**Figure 5:**
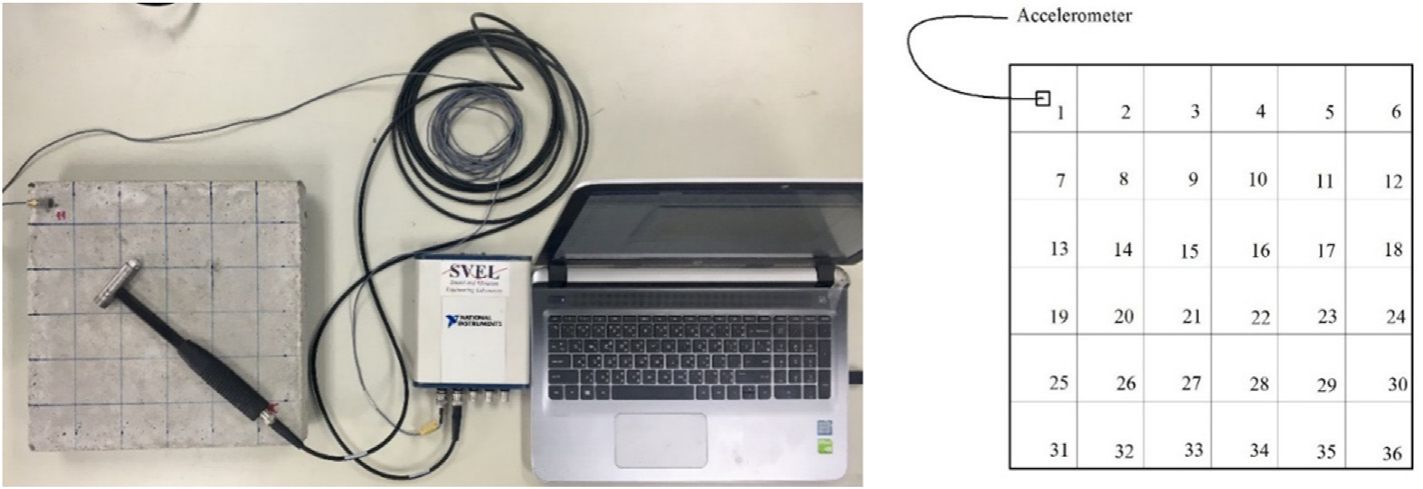
Test equipment and installation of the sensor.

The modal testing was performed to measure the mode shapes, natural frequencies, and the damping loss factors of the specimens. In the test, the surface of each specimen was divided into 36 square patches of the size 50 × 50 mm. An accelerometer was placed at the center of the top left corner patch then the impact test was performed by hitting the hammer at the center of all patches (see Figure 5). The hammer and accelerometer signals were then processed via a Matlab script to calculate the FRFs. The natural frequencies and mode shapes were determined, and the damping were calculated Eq. (5) as discussed in Section 2. The first three modes of vibration are then taken into consideration.

## 5 Results and discussion

**Figure 6:**
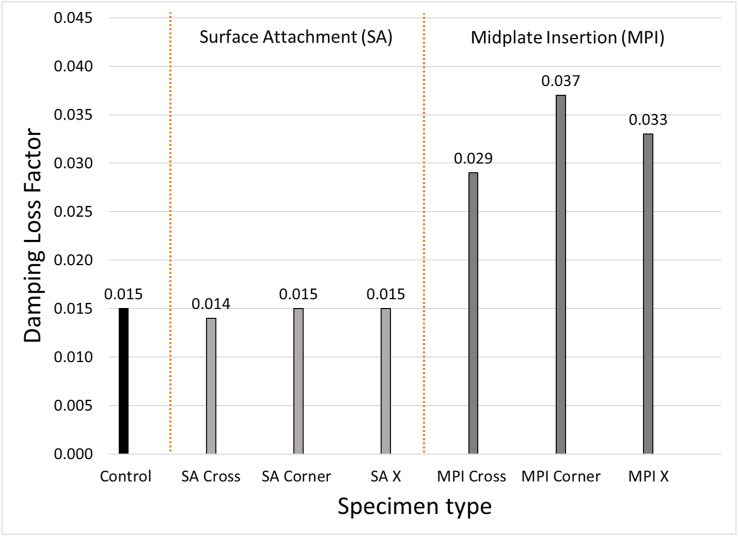
Damping loss factor of specimens in different VPS treatment conditions.

The test results are shown in Figure 6 and Table 3. It can be seen that the three first natural frequencies of the specimens with different damping treatment are approximately the same, e.g. 1,161 Hz +/- 1.5% for the 1^st^ mode, 1,909 Hz +/- 2.5% for the 2^nd^ mode, and 2,599 Hz +/- 4% for the 3^rd^ mode. This implies that adding of VPS will not significantly change the stiffness of the specimens.

**Table 3:** The damping loss factors and natural frequencies of the first three modes.

Specimen	Mode No.	Natural Frequency (Hz)	Damping Loss Factor (*η*)	Averaged Damping Loss Factor
Control	1	1,164	0.014	0.015
	2	1,974	0.016	
	3	2,781	0.014	
SA-Cross	1	1,163	0.015	0.014
	2	1,992	0.016	
	3	2,819	0.012	
SA-Corner	1	1,172	0.015	0.015
	2	2,009	0.016	
	3	2,814	0.013	
SA-X	1	1,189	0.015	0.015
	2	1,789	0.017	
	3	2,351	0.015	
MPI-Cross	1	1,091	0.030	0.029
	2	1,961	0.028	
	3	2,613	0.030	
MPI-Corner	1	1,157	0.021	0.037
	2	1,851	0.047	
	3	2,461	0.044	
MPI-X	1	1,189	0.017	0.033
	2	1,789	0.042	
	3	2,351	0.040	

The damping loss factor of the controlled specimen is use as a reference to be improved. By considering the surface constraints, the results suggested that there is no significant improvement of the damping loss factor in all specimens with the surface attachment in comparison to the control specimen. A significant improvement is found in case of mid-plate insertion. Viscoelastic materials behave similarly to viscous fluid such that they resist the shearing motion. Attaching the VPS directly to the specimens with one side able to move freely does not provide strong shearing motion and results in non-significant improvement of the damping loss factor. The shearing motion can be stronger when the constrained surface is added and should result in the improvement of the damping loss factor. This was confirmed by the experimental results in case of mid-plate insertion.

By considering the attachment pattern, the improvement can be ranked from high to low as ‘Corner’, ‘X’, and ‘Cross’ corresponding to the damping loss factor at 2.47, 2.20, and 1.90 times higher than that of the control specimen, respectively.

As mentioned in section 2 and 3, the strategy to choose pattern to attach PVS is that the pattern should cross the nodal lines as many as possible (including nodal line of higher frequencies mode shapes). The ‘Corner’ pattern seems to cross highest number of the nodal lines and following by ‘X’ and ‘Cross’. This also demonstrated by the experimental results such that the damping loss factor of mode #2 and #3 of the ‘Corner’ pattern (*η*_2_ = 0.047, *η*_3_ = 0.044) and ‘X’ pattern (*η*_2_ = 0.042, *η*_3_ = 0.040) are higher than that of the ‘Cross’ pattern (*η*_2_ = 0.028, *η*_3_ = 0.030). This is because the ‘X’ pattern crosses less numbers of nodal lines than the others.

## 6 Conclusion

This paper presents an alternative technique to improve damping loss factor by attaching viscoelastic material sheet. This will lead to some applications in sound and vibration insulation of precast concrete panels. The precast concrete specimen with combination of VPS attachment technique were prepared and tested. The results show that the mid-plate insertion of VPS is more effective in improving the damping loss factor of precast panels in comparison to the surface attachment. It was also shown that the damping loss factor can be improved from 1.93 to 2.47 times in comparison to the control specimen depending on the attachment patterns. It can be concluded that the attachment pattern also plays an important role in the improvement of the damping loss factor. The ‘Corner’ pattern was found to be the most effective in improving the damping loss factor among the others. Future investigation on the effect of VPS on sound properties of precast concrete panels is recommended to determine the relationship between damping and sound property.

## Declarations

### Author contribution statement

Khemapat Tontiwattanakul: Conceived and designed the experiments; Performed the experiments; Wrote the paper.

Jirawin Sanguansin: Performed the experiments; Analyzed and interpreted the data.

Vatanavongs Ratanavaraha & Vanchai Sata: Contributed reagents, materials, analysis tools or data.

Suchart Limkatanyu: Analyzed and interpreted the data.

Piti Sukontasukkul: Conceived and designed the experiments; Analyzed and interpreted the data; Wrote the paper.

### Funding statement

This work was supported by the National Research Council of Thailand and by King Mongkut's University of Technology North Bangkok (KMUTNB-BasicR-64-09) and by the Thailand Research Fund (RTA6280012 & MSD61I0078).

### Data availability statement

Data included in article/supplementary material/referenced in article.

### Declaration of interests statement

The authors declare no conflict of interest.

### Additional information

No additional information is available for this paper.
